# Analysis of discordant Affymetrix probesets casts serious doubt on idea of microarray data reutilization

**DOI:** 10.1186/1471-2164-15-S12-S8

**Published:** 2014-12-19

**Authors:** Andrey Marakhonov, Nataliya Sadovskaya, Ivan Antonov, Ancha Baranova, Mikhail Skoblov

**Affiliations:** 1 Research Centre for Medical Genetics, Russian Academy of Medical Sciences, Moscow 115478, Russian Federation; 2 School of Systems Biology, David King Hall, MSN 3E1, George Mason University, Fairfax, VA, 22030; 3 The Moscow Institute of Physics and Technology, Dolgoprudny 141700, Moscow Region, Russia; 4 The Russian National Research Medical University named after N.I.Pirogov (RNRMU), Ostrovityanova, 1. Moscow, 117997, Russia

## Abstract

**Background:**

Affymetrix microarray technology allows one to investigate expression of thousands of genes simultaneously upon a variety of conditions. In a popular U133A microarray platform, the expression of 37% of genes is measured by more than one probeset. The discordant expression observed for two different probesets that match the same gene is a widespread phenomenon which is usually underestimated, ignored or disregarded.

**Results:**

Here we evaluate the prevalence of discordant expression in data collected using Affymetrix HG-U133A microarray platform. In U133A, about 30% of genes annotated by two different probesets demonstrate a substantial correlation between independently measured expression values. To our surprise, sorting the probesets according to the nature of the discrepancy in their expression levels allowed the classification of the respective genes according to their fundamental functional properties, including observed enrichment by tissue-specific transcripts and alternatively spliced variants. On another hand, an absence of discrepancies in probesets that simultaneously match several different genes allowed us to pinpoint non-expressed pseudogenes and gene groups with highly correlated expression patterns. Nevertheless, in many cases, the nature of discordant expression of two probesets that match the same transcript remains unexplained. It is possible that these probesets report differently regulated sets of transcripts, or, in best case scenario, two different sets of transcripts that represent the same gene.

**Conclusion:**

The majority of absolute gene expression values collected using Affymetrix microarrays may not be suitable for typical interpretative downstream analysis.

## Background

Currently, the studies of transcriptomic landscapes in various organisms and their tissues are performed either using RNAseq or by microarrays. Even now, the latter remain the most popular and cost efficient approach for transcript profiling that is afforded by many laboratories. In particular, the most widely used microarray platform Affymetrix HG-U133A expression microarray alone provided about 1.5 millions of GEO datasets depositions available for re-analysis.

For each gene, Affymetrix arrays employ a collection of 11 to 20 very short probes; the signals from each of these probes are aggregated into a probeset-level signal. HG-U133A v2 chips include 22 283 probesets, where each probe is represented by 25 nucleotides string that matches a particular mRNA or a group of alternatively spliced RNAs. In order to minimize non-specific noise, each probeset contains not only perfectly matched probes but also mismatched ones, whose hybridization intensities are taken into account when finalized gene expression values are generated [1]. In HG-U133A microarray platform, the expression of 37% of genes is measured by more than one probeset. The discrepancies in expression of individual probesets that belong to the same gene are well-known and widespread phenomenon. Commonly, independent probeset values are averaged, and the discrepancies are underestimated, ignored or disregarded [2,3]. While the discrepancy in expression values collected in independent sets of microarray experiments may be explained by the difference in technicalities of background subtraction and normalization [4], the mismatching values collected by using two simultaneously hybridized probesets assumed to target the same transcript are more difficult to dismiss. That is why the correct annotation of probesets remains an unsolved problem.

Several attempts have been made in order to re-annotate microarrays and/or to improve data analysis workflow [5-7], with the main idea of updating an annotation of probesets by their remapping to unique target sequences, cleaning out the repeats, evaluating strand orientation [8] or analyzing the patterns of cross- and bulk-hybridization of probesets in accordance to the position of each probe on the target sequence, its GC-content, and the presence of common sequence variants [9-13]. Typically, adding the clean-out or other processing steps results in filtering out unreliable probes and/or entire probesets, thus, limiting the number of genes that may be properly analyzed in a given experiment. On the plus side, the clean-up procedures may significantly enhance the reliability of interpretation [14,15]. Sadly, these innovative steps are commonly ignored by typical microarray data processing algorithms that often contribute to either incorrect or suboptimal interpretation of expression data.

Present study aims to investigate the nature of expression value discrepancies observed for two probesets annotated to same gene.

## Results

### Discrepancies in expression values obtained using different probesets mapped to the same gene

**Figure 1:**
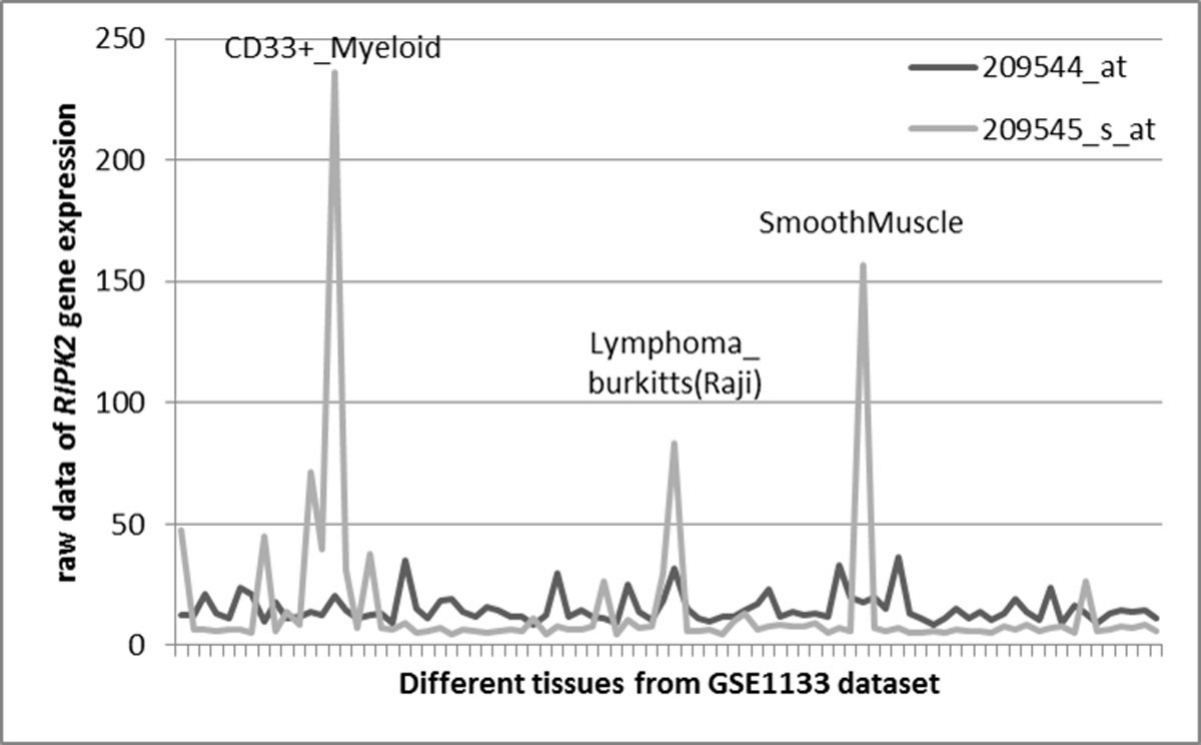
**A comparison of expression profiles obtained using two different Affymetrix HG-U133A probesets mapped to *RIPK2 *gene**.

In expression analysis by microarrays, the discrepancies in expression values obtained using different probesets mapped to the same gene are common. In case of analysis of tissue specificity, these discrepancies may be interpreted as non-congruent expression profiles obtained for the same gene using two or more probesets. As representative example, we selected human *RIPK2 *gene encoding for receptor-interacting serine-threonine kinase 2 that plays an essential role in modulation of innate and adaptive immune responses [16]. In Affymetrix HG U113A array, this gene is represented by two annotated probesets, 209544_at and 209545_s_at. The expression profiles obtained using these two probesets are incongruent. In GSE1133 [17] dataset that contains two independently obtained expression values for each of 79 different human tissues (Figure 1), the expression profile that corresponds to 209545_s_at probeset demonstrates clear upregulation in CD133-positive myeloid cells, in Burkitt's lymphoma cell line Raji and in smooth muscle, while 209544_at probeset demonstrates more or less uniform expression throughout the spectrum of tissue represented in GSE1133 panel (Figure 1).

These findings set us to find out how frequent are the expression discrepancies of this kind and to identify all reliable probeset pairs that allow extraction of similar or identical profiles. We surmised that this approach may allow us to extract a subset of expression values that correspond to true expression levels for at least a few human genes.

### Reannotation of probesets and formation of gene groups

Observations described above point that expression data obtained using single probesets may not be reliable. In the same time, the comparison of expression profiles of genes annotated by two or more probesets may serve as an internal control for validation of overall result reliability in a given microarray-based experiment.

**Figure 2:**
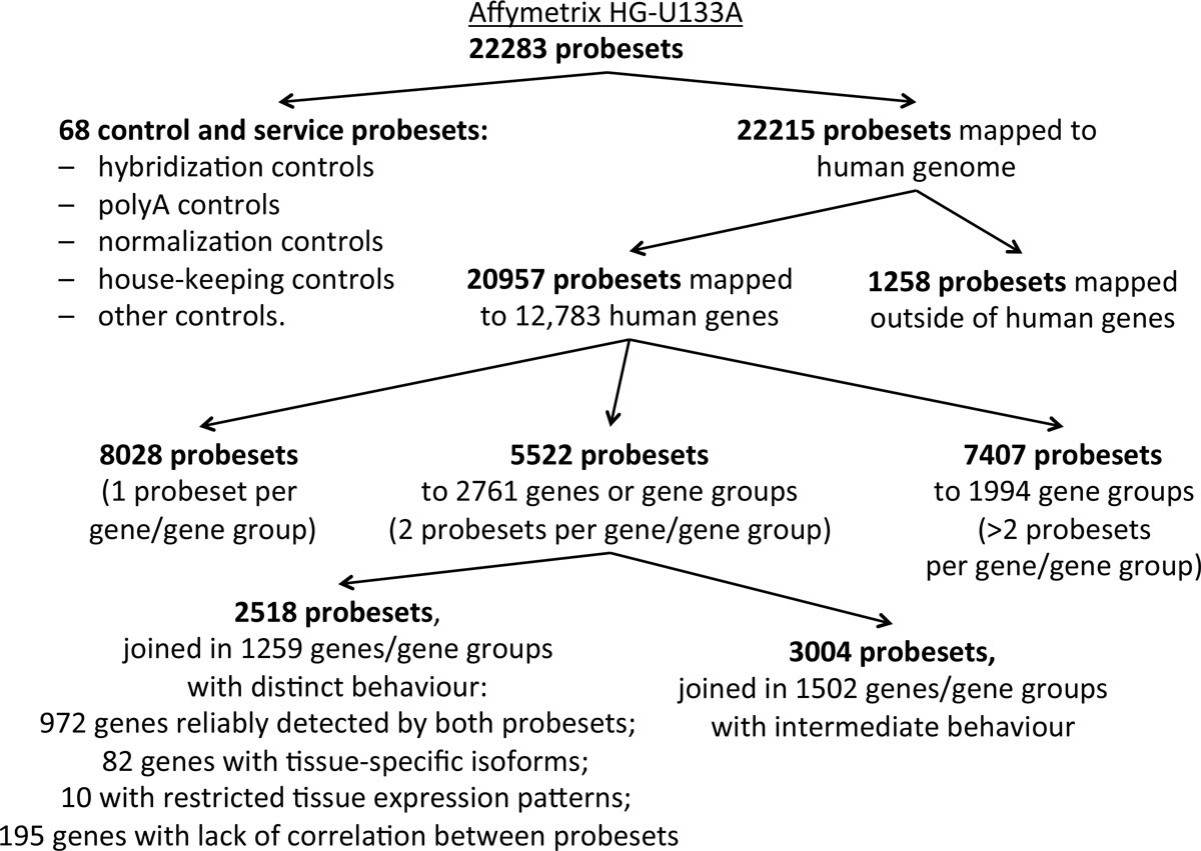
**Composition of the Affymetrix HG-U133A microarray after reannotation**.

**Figure 3:**
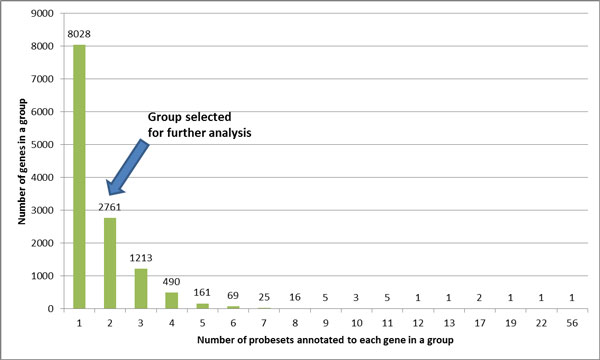
**Probeset composition per gene in Affymetrix U133A microarray after reannotation**.

Thus, for further analysis we selected only genes represented by two probesets annotated in Affymetrix HG-U133A microarray. After reannotation process (see Materials and Methods for details), the probesets were combined into *gene groups *(see details in Additional file 1 Supplementary Figure S1). The composition of Affymetrix U133A microarray platform after the reannotation is described at Figure 2. In U133A platform, the total number of gene groups that could be quantified using two different probesets is 2761, with a total of 5522 probesets (Figure 3). This number includes 87 gene groups that contain more than one gene, where all the genes are identified by same two probesets. On Additional file 1 Supplementary Figure S1, such groups are denoted as type C///D gene groups). For each analyzed gene group, the exact probeset composition could be found in Additional file 2 Supplementary Table S1.

### Correlation analysis of expression data for genes covered by two probesets

In order to identify Affymetrix probesets consistent in measuring expression of their target gene(s), an analysis of correlations was performed. Since the distribution of probeset-specific expression levels across human tissues represented in GSE1133 dataset [18] was not normal (Shapiro-Wilk test, *p *< 0.05), non-parametric Spearman's rank correlation coefficients *ρ *were calculated. To take into account that GSE1133 includes profiles for a considerable number of tissues (N = 42), we have also computed correlations using parametric Pearson's product-moment coefficient *r *which operates with absolute values of expression and is more sensitive to presence of outliers than Spearman procedure. The relative value of calculating both Spearman's and Pearson's of correlation coefficients could be illustrated using *RIPK2 *gene as an example (see Figure 1). For expression values extracted using two probesets that match to this gene, 209544_at and 209545_s_at, Pearson's correlation is 0.148, while Spearman's rank correlation is 0.144. Both types of statistics show that the correlation between expression values extracted using two different probesets is rather small (see Figure 1). However, not every gene behaved that consistently when the results of parametric and non-parametric correlation analysis were compared. In fact, this approach allowed us to differentiate human genes and gene groups into categories with specific biological properties.

**Figure 4:**
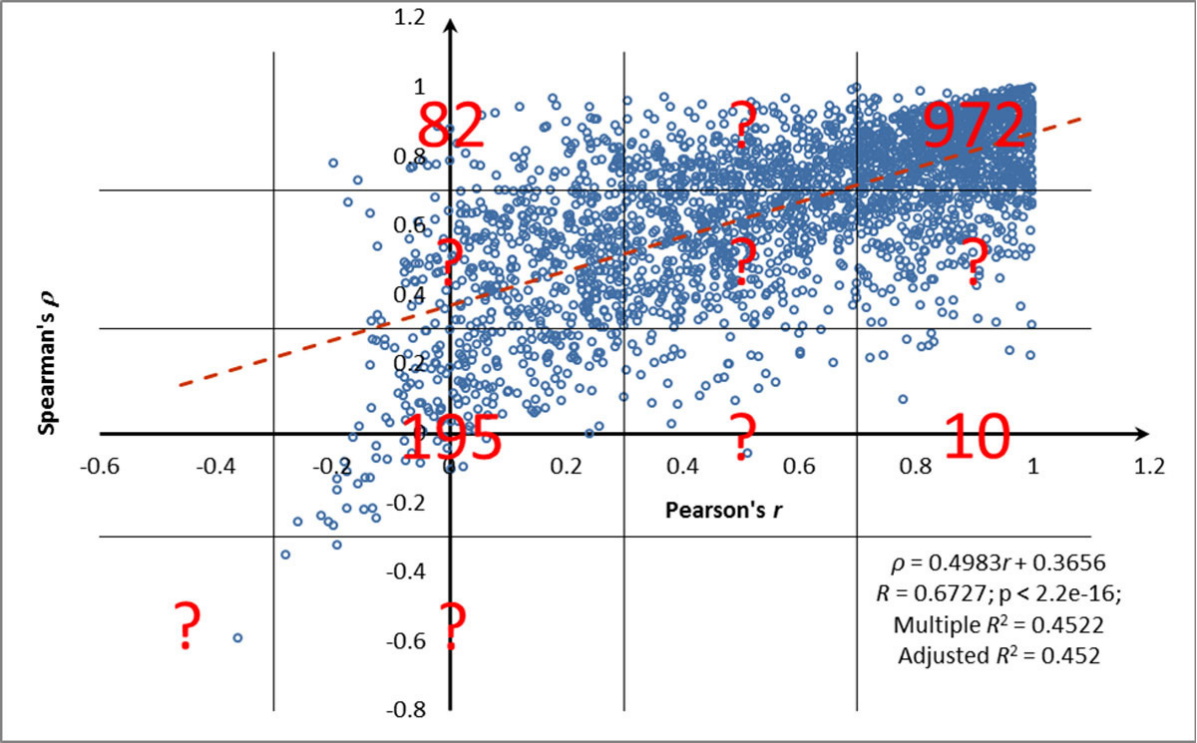
**The distribution of correlation coefficients values for a subset of genes/gene groups matched by two different probesets**. Pearson's product-moment correlation coefficient *r *is on the x-axis and Spearman's rank correlation coefficient *ρ *is on the y-axis. Each dot represents correlation between expression values obtained with two different probesets corresponding to one gene on the Affymetrix U133A microarray in GSE1133 dataset. Black lines mark the quadrants that distinguish sets of genes behaving in one or another distinct way that sets them apart from each other. The quadrants were drawn according to correlation coefficient values. Values above 0.7 and below 0.3 were considered cut-offs. Number of genes that fell into each quadrant is shown in red color. Question marks represent the areas populated by genes with less deviant behavior that is evident from milder degree of decrepancy between correlation coefficients calculated using Pearson's coefficient *r *and Spearman's coefficient *ρ*. Dark red dashed line represents linear regression line and its equation is in the bottom right corner of the plot.

For each gene that was matched by two different probesets (N = 2761), both Pearson's product-moment correlation coefficient *r *and Spearman's rank correlation coefficient *ρ *were calculated. Interestingly, it was found that two correlation coefficients show moderate positive relationship with each other (*R*^2 ^= 0.4522, *p *< 2.2e-16), as in many particular cases the parametric and non-parametric correlations dramatically differed in magnitude. A total of 1259 genes and gene groups fell into one of four extreme categories depicted on Figure 4. Thus, about 45.6% of genes matched by two probesets behaved in one or another distinct way that set them apart from each other. The list of these genes and gene groups as well as their probest composition could be found in Additional file 3 Supplementary Table S2. The remaining genes and gene groups (N = 1502) that does not belong to one or another extreme were excluded from further analysis.

### Detailed analysis of genes and gene groups that fell into extreme behavior categories

The typical way of explaining expression level discrepancies is to blame them on technical problems or uneven hybridization conditions across the chip [19]. In this study, we attempted a search for possible biological correlates that may explain incongruency of expression profiles detected by two probesets that match to the same gene.

Among four extreme categories of genes depicted on Figure 4, the first set (N = 972, or about 35.2% of genes covered by two different probesets) is the most easy to understand and accept. In this category, the correlation between expression values obtained using two different probesets and measured using both Spearman's and Pearson's procedures exceeds 0.7; let's call these genes "reliably profiled". One may extrapolate these data onto entire microarray chip, including the majority of its genes that are covered by only one probeset (N = 8028), and conclude that overall reliability of Affymetrix HG-U133A platform is about 35%. In other words, we could assert that expression profiles of approximately 35% of genes represented at this chip are measured in a reliable way.

In three other extreme categories (N = 287, or 10.4% of genes covered by two different probesets), either one or both correlation coefficients were low (< 0.3). Below we attempted to find biological, rather than technical explanation to this observation.

**Figure 5:**
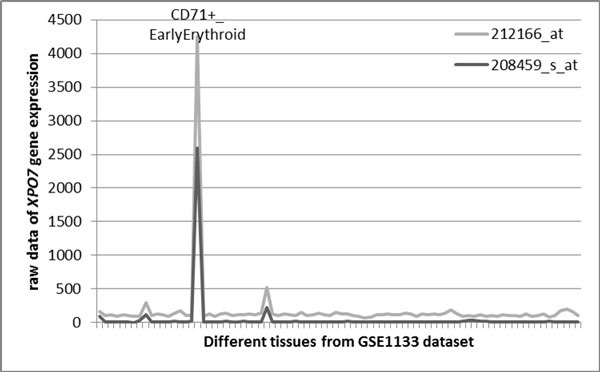
**An example of a tissue specific gene (*XPO7*) with expression patterns described by high *r *and low *ρ *correlation coefficients (set 2)**.

For the group with low Spearman's ρ but high Pearson's r coefficient (N = 10, or 0.4% of genes covered by two different probesets), the discrepancy may be explained by restricted tissue pattern of respective genes. Indeed, even in cases when expression is restricted to certain tissue, other tissues continue to produce some background expression levels. In an ideal world, the levels detected in tissues where the gene is not expressed would approximate zero. In this case, Pearson's correlation analysis correctly accounts for very similar background values in one of the tails of the distribution that is otherwise normal, while rank-based Spearman's correlation attempts to range the background values, while the distribution of these background values is close to random. The Spearman's procedure is misguided into ranking of these background values that are, in fact, should be not ranked, and produces very low correlation coefficient. This train of thoughts can be illustrated by several examples, including *XPO7 *gene (*r *= 0.998, *ρ *= 0.220) that encodes exportin 7 that shows marked expression only in CD71+ early erythroid cells [20] (Figure 5), *GPR56 *gene (*r *= 0.962, *ρ *= 0.237) encoding for G protein-coupled receptor 56 with high expression in CD56+ natural killer cells [21] and thyroid gland [22], and *FXYD3 *(*r *= 0.745, *ρ *= 0.222) that encodes for FXYD domain containing ion transport regulator 3 that demonstrates strong expression in colon only [23]. All together, this logic indicates that in case of genes with restricted pattern of expression, the correlations should be measured using Pearson's rather than Spearman's procedure as only Pearson's coefficients adequately reflect the nature of tissue-specific gene expression. Figure 5 visually demonstrates that expression patterns described by *XPO7 *probesets 208459_s_at and 212166_at are, indeed, in good correspondence to each other.

**Figure 6:**
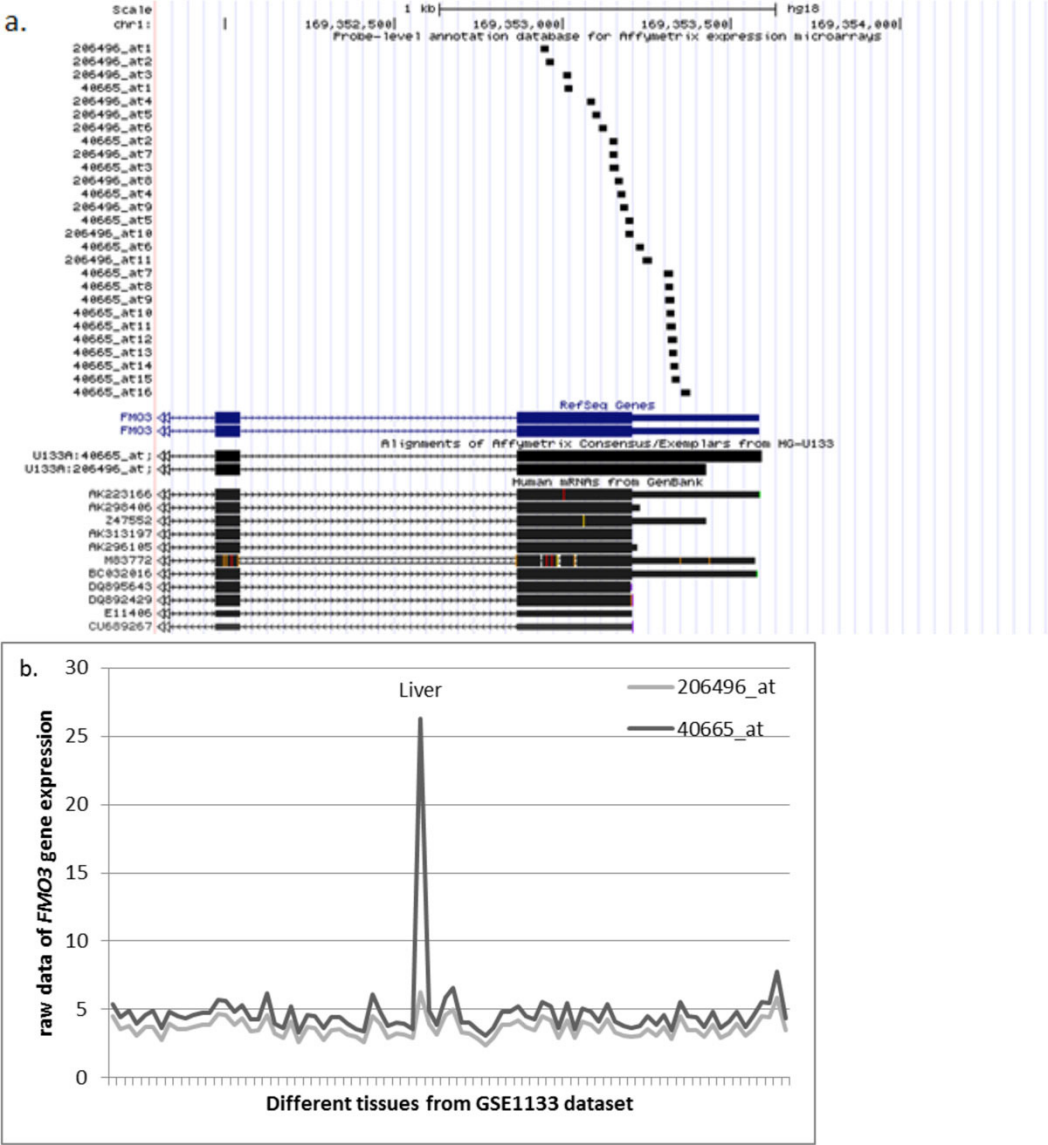
**An example of a gene (*FMO3*) with tissue-specific isoforms with expression patterns described by low *r *and high *ρ *correlation coefficients (set 3)**. a. The mapping of the probesets 40665_at and 206496_at to the genomic location of *FMO3 *gene according to PLANdbAffy custom track in UCSC Genome Browser. b. Expression profile of the *FMO3 *gene in tissue panel of the GSE1133 dataset.

For the group with high Spearman's *ρ *but low Pearson's *r *coefficient (N = 82, or 3% of genes covered by two different probesets), the discrepancy of correlation coefficients may be explained by the presence of tissue-specific isoforms. When the gene exhibits an expression profile with specific RNA isoform spiking in some but not other tissues, Spearman's correlation remains high because it ranks the expression values in tissues with overexpressed transcript variant on top of the list of values, while all other tissues are being shifted to the bottom while keeping their order. On the other hand, Pearson's coefficient slips to the lower output due to its sensitivity to a spike in absolute values of expression brought by strong up-regulation of expression signal in one probeset produced in one or a few tissues even though in all other tissues the signals remain similar to those generated by another probeset. This hypothesis could be investigated directly by evaluating whether two different probesets for a gene generating high Spearman's *ρ *but low Pearson's *r *coefficients predominantly maps to two different exons or alternative 5′- or 3′-UTR of the same gene. To assess this, we analyzed the mapping patterns of individual Affymetrix probes systematically described in alignments in PLANdbAffy database [24]. In a majority of cases, our assumptions were correct. For example, an expression of the flavin containing monooxygenase 3 encoding gene *FMO3 *(*r *= 0.691, *ρ *= 0.989) is commonly described as restricted to liver [25,26]; this fact is correctly reflected by hybridization pattern of probeset 40665_at, but not by that of probeset 206496_at. Both probesets are located in the terminal exon of the gene (Figure 6), with probeset 40665_at being shifted toward 3′ end of the transcript. An analysis of GenBank records of various transcripts produced by *FMO3 *locus (Figure 6a) demonstrates that the bulk of mRNAs terminate shortly before the location of the majority of the probes comprising the probeset 40665_at. It seems that the liver-specific expression of *FMO3 *is restricted to its mRNA isoform with longer 3′-UTR only, while the shorter mRNA isoform is expressed ubiquitously with relatively low abundance. Another possible explanation may be an existence of liver-specific RNA associated with 3′-UTR of *FMO3 *gene that is expressed independently of its major mRNA isoform; a number of RNAs that fits this description have recently been described by Mercer as *trans*-acting uaRNAs [27].

**Figure 7:**
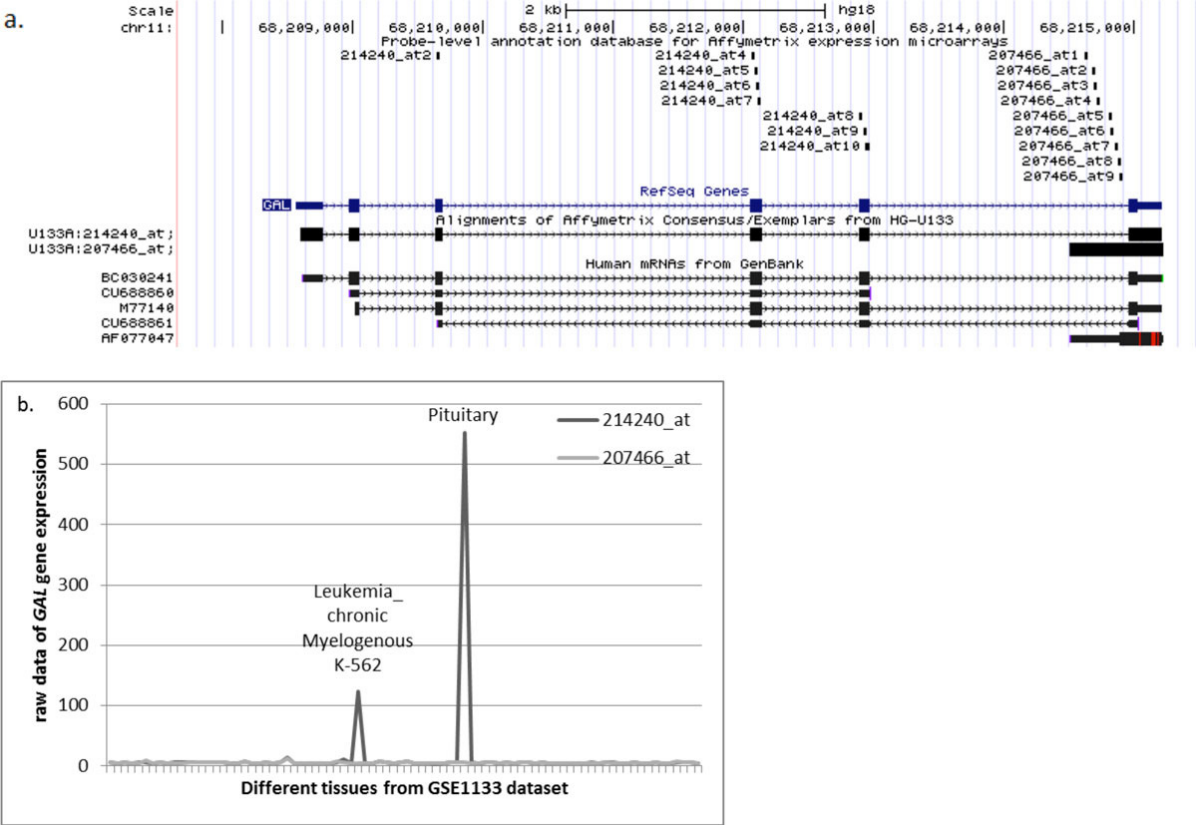
**An example of a gene (*GAL*) with expression patterns described by low *r *and high *ρ *correlation coefficients (set 3)**. a. The mapping of the probesets 2142405_at and 207466_at to the genomic location of GAL gene according to PLANdbAffy custom track in UCSC Genome Browser b. Expression profile of the GAL gene in tissue panel of the GSE1133 dataset.

Another example is galanin/GMAP prepropeptide encoding gene *GAL *(*r *= 0.057, *ρ=*0.914) characterized by strong expression in pituitary gland [28]. This gene is associated with two probesets, 207466_at and 214240_at. The probeset 214240_at is distributed among three internal coding exons of *GAL*, while probeset 207466_at aligns to an intron adjacent to the last exon of GAL and possibly reflects an expression of an alternatively spliced isoform of its last exon that corresponds to the only one mRNA in GenBank, AF077047 (Figure 7). Therefore, the hybridization pattern of these two probesets should be interpreted according to that of the probeset 214240_at as expression restricted to the pituitary and CML cell line sk-562, while probeset 207466_at reports ubiquitous expression that does not exceed the background level and possibly should be ignored.

There is no easy explanation for the discordant expression values observed for genes or gene groups with low correlation values revealed by both Spearman's and Pearson's coefficient (N = 195, or 7.1% of genes covered by two different probesets). A very good example of this kind is a *RIPK2 *gene (*r *= 0.148, *ρ *= 0.144) described at the Figure 1. The remarkable discrepancy in the expression patterns derived from two probesets that match to the same gene may be due either to technical or methodological problems, but also to some unknown biological reasons.

### An influence of the quality of probes alignment on the concordance of expression patterns

To find out whether misalignment of individual probes that comprise particular probesets could cause the discrepancy in the expression patterns, we downloaded and explored genomic alignments of individual probes mapped to human genome in PLANdbAffy database [24]. In his paper, Nurtdinov *et al*. classified all Affymetrix probes into four classes and assigned a color to each class. In his classification, the "green" probes are most reliable; these probes satisfy the following three conditions: (i) the probe is aligned to the target gene with no mismatches, (ii) there are no matches of the probe to other genes and (iii) there are no perfect alignments of the probe to any non-coding region. The "yellow" probes match the criteria (i) and (ii) but not (iii). The "red" probes are the perfect match to the target gene and to at least one other gene with no more than one mismatch. Finally, the "black" probes are aligned to the target gene with at least one mismatch [24]. We adopted color-based classification of the probes described above to augment our own reannotated file that described probesets that comprise Affymetrix HG-U133A microarray with percentages of individual probes that belong to each PLANdbAffy color class [24] (see Additional file 2 Supplementary Table S1). Notably, both probesets to *RIPK2 *gene (Figure 1) are almost exclusively comprised of "green" probes and nearly perfectly map its cognate gene. The exclusion being the probe 209545_s_at_5 that matches *RIPK2 *gene in its 20 out of 25 nucleotides, thus, being a "black" probe.

In its formidable effort, Nurtdinov *et al*. comprised the database that hosts the genomic alignments for all individual Affymetrix probes, while making no attempt to find out whether the quality of the probes may affect the accuracy of expression profiling. In fact, it seems logical to conclude that the quality of the probes, indeed, defines the quality of microarray output. However, in present work, we demonstrate that this is not the case.

**Figure 8:**
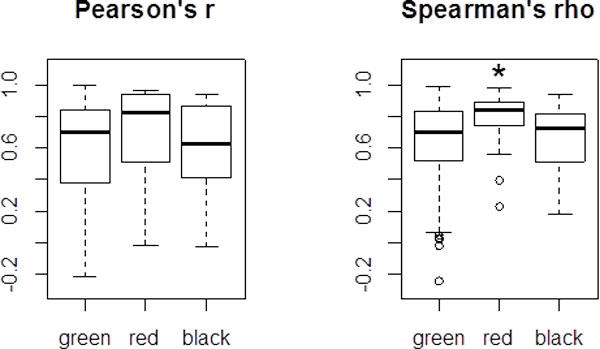
**The boxplot of Pearson's (left) and Spearman's (right) correlation coefficients within the "color-coded" groups of genes matched by two different probesets of equivalent quality**. Only "green", "red" and "black" groups of genes are shown, as "yellow" group contains no genes. In each color-coded group, bold horizontal line corresponds to a median, while box denote 25-75% range. The results of Kruskal-Wallis one-way ANOVA test are in a right top box of plot. Star indicates significant difference by Mann-Whitney *U *test (*p *< 0.05, Bonferroni correction).

To prove this point, we selected the genes covered by two different probesets with the same quality of the comprising probes as assessed by classification of Nurtdinov *et al*. [24]. A total of 340 genes satisfied this criterion, with 297 genes falling into "green" group, 27 genes - into "red" group, and 16 genes - in "black" group, while no gene with two different probesets fitting "yellow" criterion were found. (Additional file 4 Supplementary Table S3). Using these perfectly matched groups of genes, we analyzed whether the quality of probeset affects the correlation between expression profiles obtained using two independent probesets. Speaking generally, the higher the quality of individual probes that comprise two probesets is, the higher correlation between expression profiles obtained using these two probesets should be observed. Surprisingly, only Spearman's correlations were significantly different among the color groups (*p *< 0.005, by Kruskal-Wallis one-way ANOVA test), while Pearson's correlations demonstrated large standard deviation and were not able to differentiate the groups (Figure 8).

**Figure 9:**
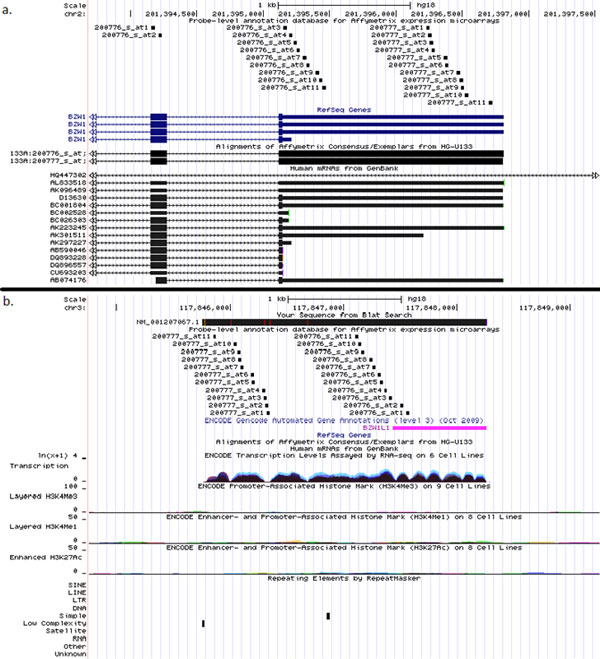
**An example of a gene (*BZW1*) with pseudogene (*BZW1L1*) which expression patterns described by high *r *and high *ρ *correlation coefficients**. a. The mapping of the probesets 200776_s_at and 200777_s_at to the genomic location of BZW1 gene according to PLANdbAffy custom track in UCSC Genome Browser. b. *BZW1L1 *pseudogene as well as alignment of probe sets annotated to its parent *BZW1 *gene (picture view from UCSC Genome Browser). For clarity, the BLAT search with NM_001207067.1 (transcript variant 1 of the parent *BZW1 *gene) as a query is also shown.

The most surprising conclusion that could be drawn from this analysis is that expression profiles derived from the hybridization patterns of the "red" probesets that map to the same gene but also align to at least one other gene with no more than one mismatch, on average, better match each other that these obtained using "green" probesets that perfectly match cognate gene. One possible explanation to this situation is that these "red" probes are aligned to both the target gene and its pseudogene(s) or to the whole family of conserved paralogs. For example, basic leucine zipper and W2 domains 1 encoding gene *BZW1 *[29] is annotated by two probesets, 200776_s_at and 200777_s_at, with closely matching hybridization patterns in human tissues (*r *= 0.899, *ρ *= 0.756) (Figure 9a). While gene *BZW1 *is located on chromosome 2q, its two processed pseudogenes *BZW1P1 *and *BZW1P2 *are found on 3q26.31 and 3q13.31, respectively. These pseudogenes display 99.6% and 94.3% identity to the longest mRNA encoded by *BZW1 *(NM_001207067.1), while overlapping 2983 nt and 2505 nt out of 3400 nt, respectively. Due very high level of homology, both BZW1 probesets align both to its cognate gene and its pseudogenes. As a rule, processed pseudogenes lack their own promoters and may be transcribed only from the promoters of neighboring genes or repeats; in these cases expressed pseudogenes are unlikely to preserve expression pattern of its parental transcript [30]. According to high concordance in the expression of two "red" probesets observed in GSE1133 tissue profiles, it seems that two *BZW1 *pseudogenes are not transcribed and that both probesets quantify the expression of only one gene, *BZW1*. This observation is in accordance with the ENCODE data showing no activity of the *BZW1 *pseudogenes' promoters in all tested cell lines (Figure 9b). Thus, "red" probesets 200776_s_at and 200777_s_at should not be, in fact, classified as "red", but rather "green". It should also be noted that expression profiling congruency dissection approach for genes represented by "red" and "green" probesets could be useful for global identification of expressed and silent pseudogenes in available microarray data.

Another reason for higher correlation between gene expression profiles derived from the hybridization patterns of "red" probesets could be the alignment of these probes to a whole family of conserved paralogs. A good example of such situation is a highly conserved family of *NOMO1*///*NOMO2*///*NOMO3 *genes with an average identity between transcripts of 95-100%. *NOMO *genes originate from a genomic duplication at least 78 Mb in size that took place in 16p12.3-p13.1. Both *NOMO*-specific probesets, 217225_x_at and 221853_s_at, are annotated to all *NOMO *genes and, therefore, fall into "red" category. High degree of correlation between gene expression profiled derived from the hybridization patterns of those probesets (*r *= 0.958, *ρ *= 0.962) may be explained by similarly high correlation of expression profiles of all *NOMO *genes that share their regulatory features that were duplicated all together with the genes itself.

**Figure 10:**
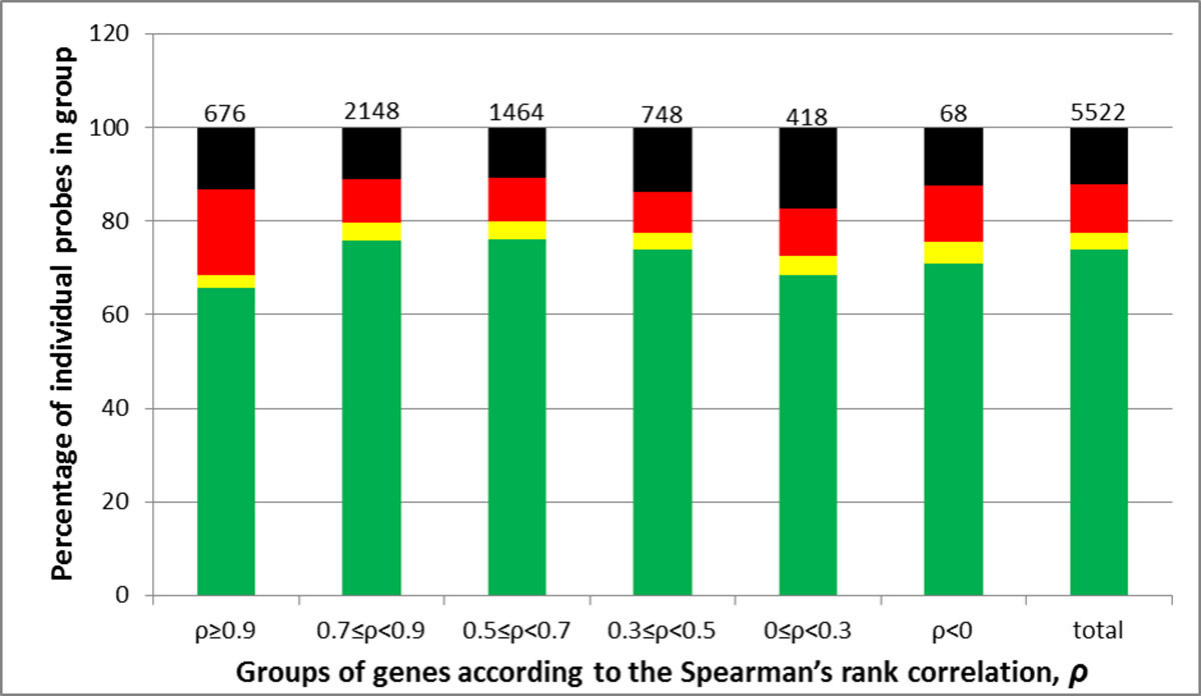
**Barplot of probes quality content in different subsets of genes/gene groups matched by two different probesets depending on correlation strength**. Statistical analysis aimed at estimation of relative influence of the probeset quality on the strength of correlation between expression profiles obtained with two different probesets annotated to one gene. Numbers above the bars represents number of probesets of each category.

These examples demonstrate that the quality of individual probes that comprise probesets does not explain the discrepancy in expression profiles obtained by hybridizing these probesets. As we realize that this kind of conclusion should be tested as rigorously as possible, we also tested for possibility that our test sample of genes was not representative of the whole set of genes. Indeed, choosing to compare only genes matched by two probesets of exactly the same quality, we dramatically decreased the size of the sample by selecting only 27 pairs of gene-matched "red" probesets, and 16 pairs of gene-matched "black" probesets, while ignoring "yellow" probesets we could not find being paired. Hence, we tried to estimate relative influence of the probeset quality on the strength of correlation between expression profiles obtained with two different probesets annotated to one gene. If the influence is substantial, than as higher the correlation between expression profiles derived from hybridization patterns of two probesets, the better the quality of individual probes should be. Unexpectant, the strength of correlation was not related to the probesets quality (Friedman ANOVA test p-value = 0.9164; Kendall coefficient of concordance = 0.9510204; Figure 10).

### Is the derivation of real-life tissue expression patterns from microarray data possible at all?

To tackle this Holy Grail problem, for each gene covered by two different probesets (N = 2761) we compared the means (that correspond to the average expression level of a particular gene between different tissues) and coefficients of variation (that reflect the variance of expression for a particular gene between different tissues and could indicate whether certain gene is ubiquitously expressed or not) between both probesets. This type of analysis was previously executed for genes covered by three or more probesets in [31]; in that study Jaksik *et al*. suggested similarly designed search for the outliers among probesets annotated to same gene with subsequent elimination of such a probe set from further analysis. However, this approach is applicable with confidence only to genes annotated with more than two probesets. Here we took approach of Jaksik *et al*. a bit further, by suggesting that we may trust that two independent gene-specific probesets to report correct expression profiles if these probesets show comparable means and coefficients of variation. In other words, even if expression profiles derived from hybridization patterns of these probesets demonstrate lower than expected correlation to each other, we may hope for a salvation by averaging the values produced by each probeset, and treating the cumulative value as true expression value for given gene; thus, we would justify the procedure that is typically applied in garden-variety microarray analysis pipelines, especially in gene-based approaches like coexpression network analysis and gene set enrichment analysis [32-35].

**Figure 11:**
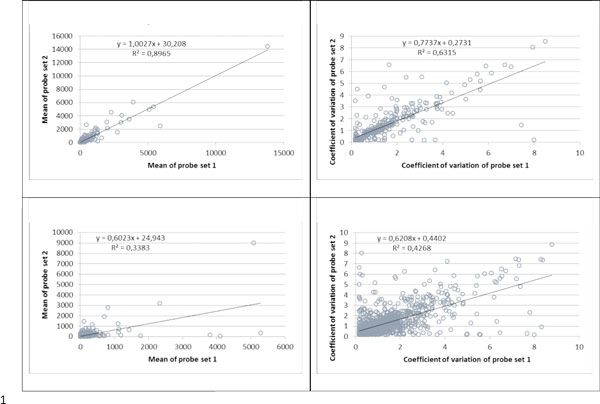
**Plot of means and coefficients of variation of expression data obtained with different probe sets annotated to one gene with Spearman's correlation *ρ *≥ 0**.9 (upper panel) and 0.7 ≤ *ρ *< 0.9 (lower panel). Each dot represents means of expression data (left part) or coefficients of correlation (right part) obtained with two probesets corresponding to one gene on the Affymetrix U133A microarray in GSE1133 dataset (probe set 1 on the x-axis and probe set 2 on the y-axis). Black line represents linear regression line and its equation is in the top left corner of plot. See also Additional file 5 Supplementary Fig S4.

According to their Spearman's correlation coefficients, the genes matched by two probesets were divided into several subgroups. Again, in a contrary to our best wishes, the strongest matches between both means and coefficients of variation were almost exclusively found only within the subgroup of genes covered by the probesets that already produce an agreement in their expression profiles (338 genes with *ρ *≥ 0.9 and 1074 cases with 0.7 ≤ *ρ *< 0.9, Figure 11) while other subgroups showed significantly lower relationship (see also Additional file 5 Supplementary Table S4). In other words, when two different probesets that match the same gene fail to show strong correlation in respective expression profiles, they also tend to demonstrate substantial mismatch in means and coefficients of variation of expression values reported.

## Discussion

Even advent of RNAseq cannot defy current reality: expression microarrays remain an important tool that allows one to discern gene expression profiles at a genome scale. Moreover, the adoption of the Minimum Information About a Microarray Experiment (MIAME) standard and the establishment of public repositories for microarray data, especially Gene Expression Omnibus (GEO) and ArrayExpress set the stage for gene expression data sharing and reuse (see [36] for review). Intuitively, due to stoichiometric nature of sequences hybridization, the signal rendering should be directly proportional to the concentrations of cognate RNA molecules in tested samples. Moreover, the design of expression microarrays, with several individual probes corresponding to each single gene and comprising the "probeset" should guarantee intrinsic robustness of the detection and the consistency of the expression profiles produced. Nevertheless, in the vast majority of experiment, different probesets corresponding to the same gene differ in the levels of the signal they generate, thus, demonstrating discordant expression profiles. This raises the question of whether expression microarray data are reliable at all. This is not an idle question, as microarrays are used not only in research labs, but also in the process of the discovery of diagnostic biomarkers and in the screening for novel medical drugs.

An initial goal of our study was to develop the criteria for selection of the most reliable probesets. Instead, in process of this analysis, the study focus was shifted to investigation of overall reliability of the data we could possibly obtain from expression microarray. In this work, we evaluated the outputs of widely used Affymetrix HG-U133A platform. To compare the signals produced by various probesets that comprise this array, we selected a dataset that profiled expression levels in 84 different human tissues. First, in order to identify possible biological reasons that could cause the discrepancy of expression profiles obtained using two probesets that map to the same gene, we performed correlation analysis of these expression profiles and found that the these correlations aid in classifying the genes represented by two probesets into distinct functional subgroups, including one enriched by genes expressed only in specific tissue(s) and another that maps onto alternative transcripts produced by the same locus. Moreover, we have found that the analysis of expression patterns revealed by the pairs of same-gene probesets aligned both to the gene and its pseudogene(s) may help to differentiate between expressed and non-expressed pseudogenes.

In further attempt to confirm commonly discussed notion that the "quality" of the probes that comprise probesets directly affect the expression data outputs, we have also analyzed the locations of individual probes and their quality classes. Contrary to our expectations, we could not confirm the relationship between probe sets "quality" and the concordance of expression profiles obtained with the same-gene probesets matched by their quality. Hence, commonly observed differences in expression profiles obtained with different probesets that match the same gene are not due to low quality of the probesets mapping but to something else. Most commonly cited reason for such inadequate results is the technical errors [19,37].

Finally, in order to investigate the reliability of expression signals obtained with different probesets that match the same gene we have compared the means and coefficients of variation of expression values obtained with these probesets. To our surprise, we observed that only genes that display almost perfect correlation between two expression profiles that correspond to these probesets (ρ ≥ 0.9, Figure 11) display comparable distributions of absolute expression values produced by both probesets. The subgroups of genes with lower degrees of correlation between expression profiles show significantly lower consistency in terms of absolute values of expression. Most likely explanation to this phenomenon is that, indeed, these probesets report differently regulated sets of transcripts, or, in best case scenario, two different sets of transcripts that represent the same gene. This observation indicates that at least 65% of the absolute gene expression values collected using Affymetrix microarrays cannot be utilized for typical interpretative downstream analysis.

## Conclusion

MIAME 2.0 project called for reutilization of microarray data to produce more relevant and robust results. Unfortunately, our study led us to the conclusion that even the most reliable probeset-based expression microrrays fail to produce accurate reflection of the expression profile for individual transcripts. Hence, the reutilisitaion of microarray datasets may be possible only through analyzing individual probes or cleaned up sets of probes [38,39] that went through extensive validation by both genome alignments and by the dissection of reference sets of microarray profiles.

## Materials and methods

The test Affymetrix HG U133A dataset GSE1133 was downloaded from BioGPS database [18]. The Affymetrix HG-U133A platform was selected because of its popularity and its design centered on annotated probesets that match validated human genes [24].

Reannotation of Affymetrix HG U133A was performed in two steps that started with Affymetrix annotation build 32 (#%netaffx-annotation-netaffx-build = 32). General overview of the reannotation process is described in the Additional file 1 Supplementary Figure S1. First, the probesets annotated to same gene were grouped together. In example presented at the Additional file 1 Supplementary Figure S1, probesets 2 and 3 were classified as Group B because they both were annotated to gene B. Even in original Affymetrix annotantion, a total of 583 probesets annotated to several genes simultaneously and denoted as C///D where C and D are two different genes (see probeset 4 at Additional file 1 Supplementary Figure S1 as an example). For convenience of the following analyses, when the probesets were defining two or more genes at the same time, this group of genes was classified as novel, combinatorial expression group. Sometimes these two genes could be also covered by additional, truly differentiating probesets, such as probesets 5 and 6 in our example. In these cases, each of the genes was assigned to its own gene label that could be profiled only by differenting probeset, but not by the probeset that could be used to profile two or more genes at the same time. For each gene, or gene group, the amount of differentiating probesets was calculated.

During second step of reannotation, all probesets were combined into 'gene groups' in a following manner: C///D ∪ C ≡ C///D (the union of C///D and C is defined as C///D gene group). According to this procedure, probe sets 4, 5 and 6 were grouped in gene group 'C///D' (see Additional file 1 Supplementary Figure S1). Notably, original attribution of each probeset to gene was preserved throughout the reannotation procedure. Thus, annotation information about each probeset was only updated and extended.

In each individual probeset, all probes were individually aligned and mapped to human genome using PLANdbAffy database and custom track in UCSC Genome Browser [24].

Microsoft Excel 2010 (Microsoft Corporation) was used to perform the correlation analysis. Other statistics were computed using R [40].

## Competing interest

The authors declare that they have no competing interest.

## Authors' contributions

AM has made contributions to conception and design of reannotation, acquisition of data, statistical analysis and interpretation of data, as well as drafting the manuscript. NS has developed custom-made C++ script for reannotation of Affymetrix microarray. IA has been involved in critical revision of the manuscript. AB conceived of the study, and participated in its design and coordination and helped to finalize the manuscript. MS has made contributions to conception and design of the study as well as interpretation of data. All authors read and approved the final manuscript.

## Supplementary Material

Additional file 1 **Supplementary Figure S1**. Reannotation pipeline Click here for file

Additional file 2 **Supplementary Table S1**. Reannotation of Affymetrix HG-U133A microarray Click here for file

Additional file 3 **Supplementary Table S2**. Sets of genes classified according to correlation analysis Click here for file

Additional file 4 **Supplementary Table S3**. A list of one color probes genes Click here for file

Additional file 5 **Supplementary Table S4**. Means and coefficients of variation of different two-probesets gene groups according to Spearman's correlation analysis Click here for file

## Declarations

Publication of this article has been funded by the BGRS\SB-2014 Organizing Committee.

This article has been published as part of *BMC Genomics *Volume 15 Supplement 12, 2014: Selected articles from the IX International Conference on the Bioinformatics of Genome Regulation and Structure\Systems Biology (BGRS\SB-2014): Genomics. The full contents of the supplement are available online at http://www.biomedcentral.com/bmcgenomics/supplements/15/S12.
