# Supplementary material for: Analysis of discordant Affymetrix probesets casts serious doubt on idea of microarray data reutilization
Source: BMC Genomics. 2014 Dec 19;15(Suppl 12):S8. doi: 10.1186/1471-2164-15-S12-S8 (PMC4303952; doi:10.1186/1471-2164-15-S12-S8)
Supplement: Additional file 1 — Supplementary Figure S1. Reannotation pipeline [file 1471-2164-15-S12-S8-S1.pptx]

## Slide 1
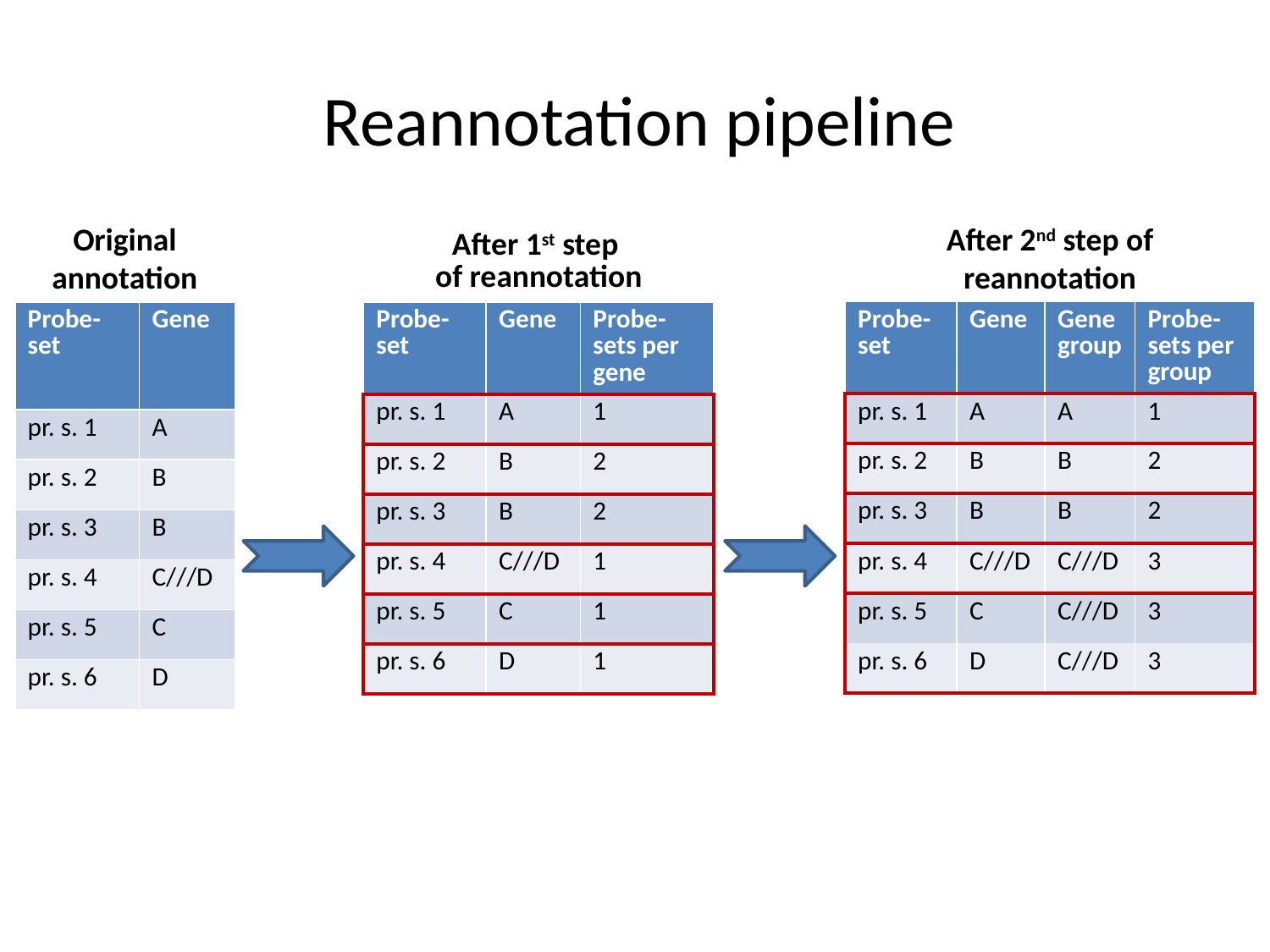

# Reannotation pipeline
After 1st step
of reannotation
Original annotation
After 2nd step of reannotation
| Probe-set | Gene | Gene group | Probe-sets per group |
| --- | --- | --- | --- |
| pr. s. 1 | A | A | 1 |
| pr. s. 2 | B | B | 2 |
| pr. s. 3 | B | B | 2 |
| pr. s. 4 | C///D | C///D | 3 |
| pr. s. 5 | C | C///D | 3 |
| pr. s. 6 | D | C///D | 3 |
| Probe-set | Gene |
| --- | --- |
| pr. s. 1 | A |
| pr. s. 2 | B |
| pr. s. 3 | B |
| pr. s. 4 | C///D |
| pr. s. 5 | C |
| pr. s. 6 | D |
| Probe-set | Gene | Probe-sets per gene |
| --- | --- | --- |
| pr. s. 1 | A | 1 |
| pr. s. 2 | B | 2 |
| pr. s. 3 | B | 2 |
| pr. s. 4 | C///D | 1 |
| pr. s. 5 | C | 1 |
| pr. s. 6 | D | 1 |
