# Supplementary material for: Analysis of discordant Affymetrix probesets casts serious doubt on idea of microarray data reutilization
Source: BMC Genomics. 2014 Dec 19;15(Suppl 12):S8. doi: 10.1186/1471-2164-15-S12-S8 (PMC4303952; doi:10.1186/1471-2164-15-S12-S8)
Supplement: Additional file 5 — Supplementary Table S4. Means and coefficients of variation of different two-probesets gene groups according to Spearman's correlation analysis [file 1471-2164-15-S12-S8-S5.docx]

| Correlation strength | Mean 1 vs mean 2 | Coef. of var. 1 vs coef. of var. 2 |
| --- | --- | --- |
| ρ>=0.9 (338 cases) | 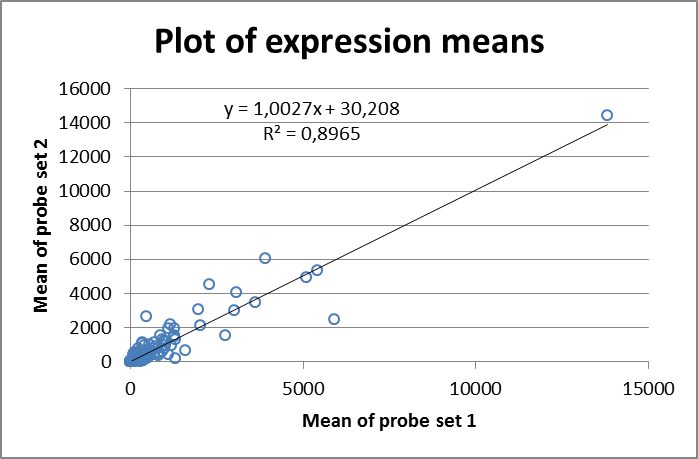 | 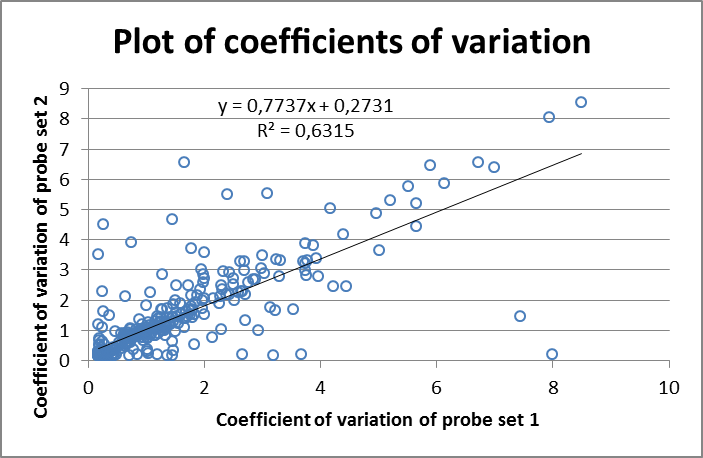 |
| 0.7=<ρ<0.9 (1,074 cases) | 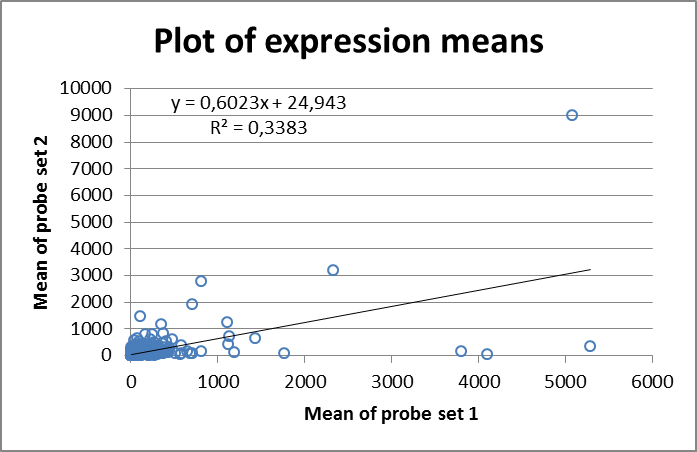 | 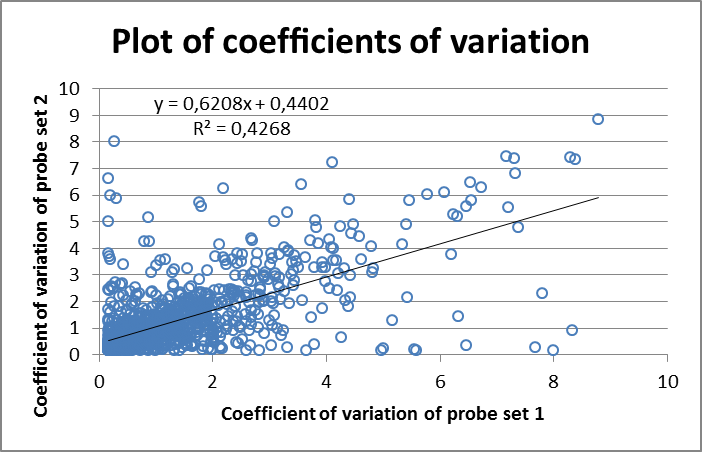 |
| 0.5=<ρ<0.7 (732 cases) | 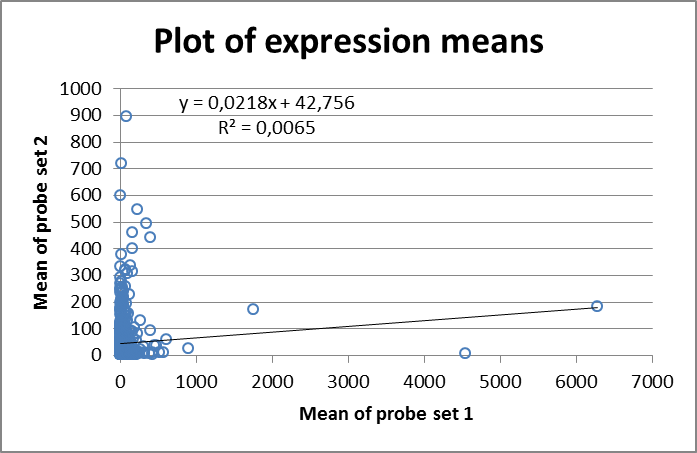 | 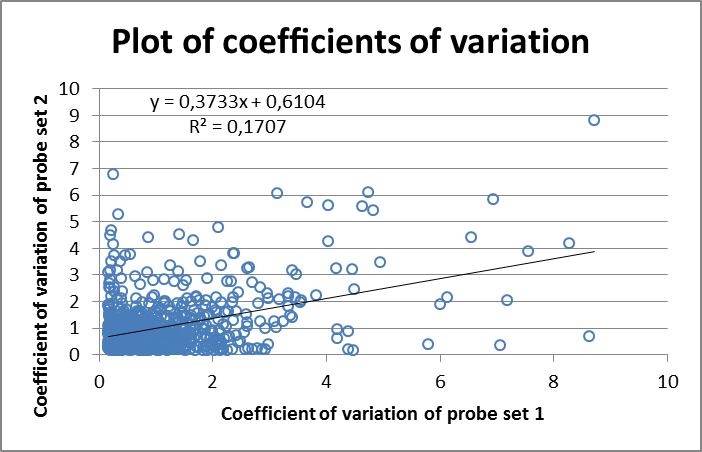 |
| 0.3=<ρ<0.5 (374 cases) | 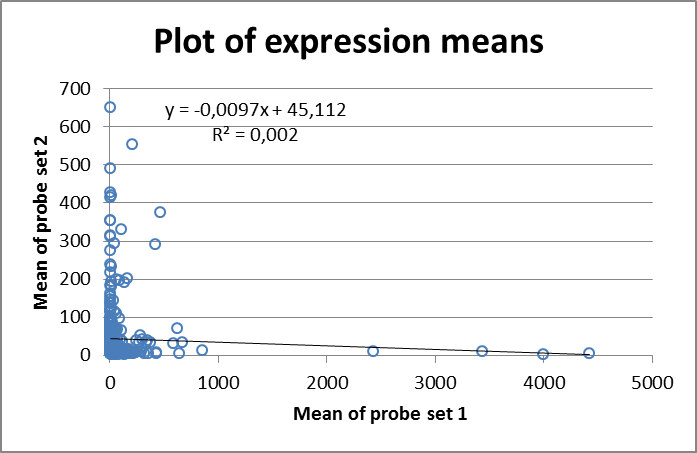 | 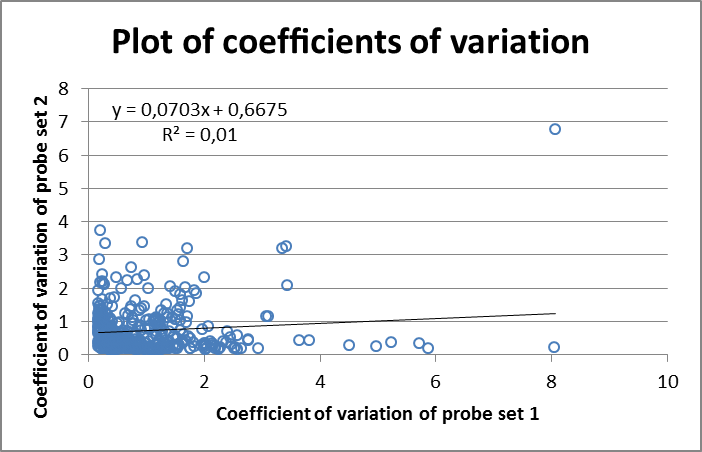 |
| 0=<ρ<0.3 (209 cases) | 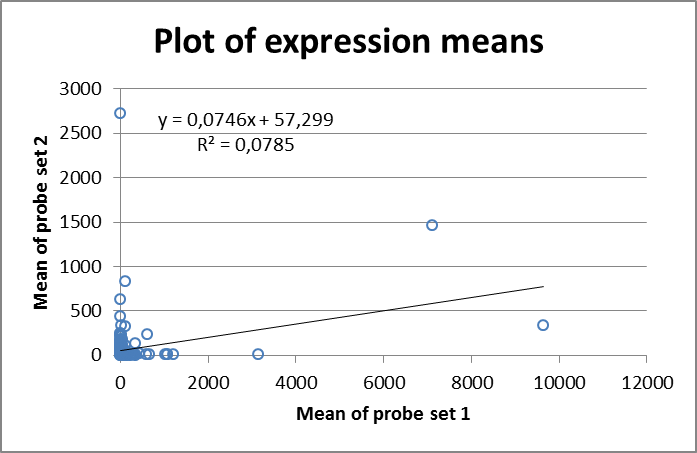 | 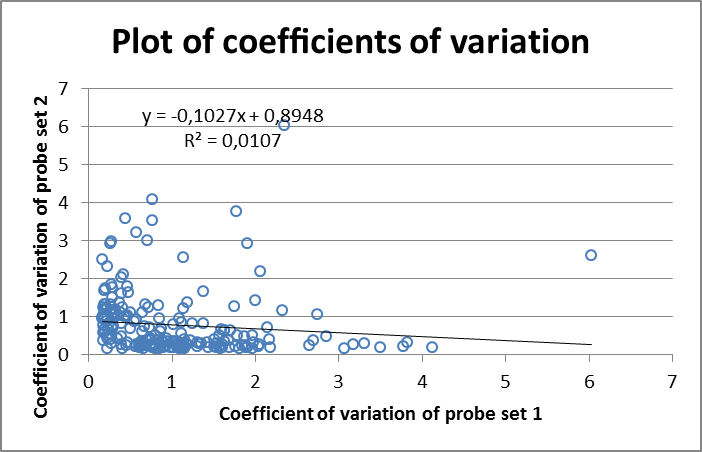 |
| Ρ<0 (34 cases) | 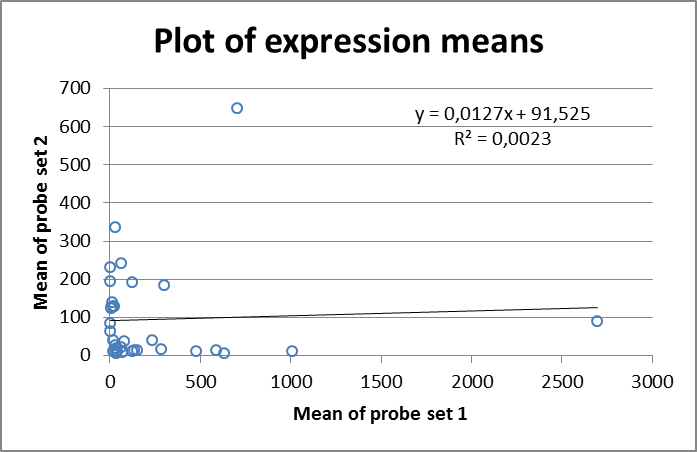 | 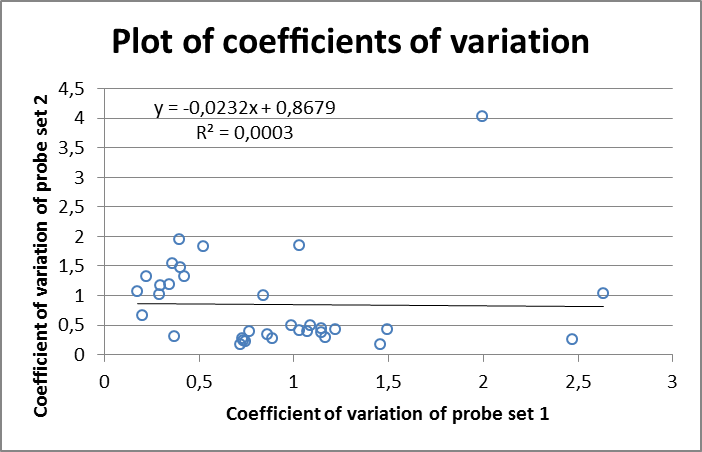 |
| Total:  2,761 cases | 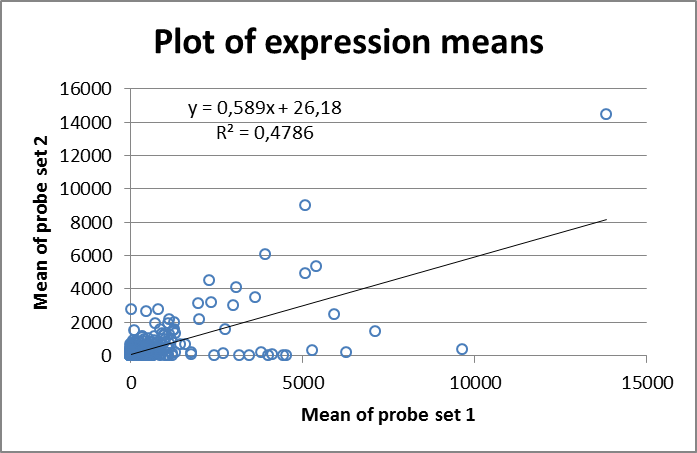 | 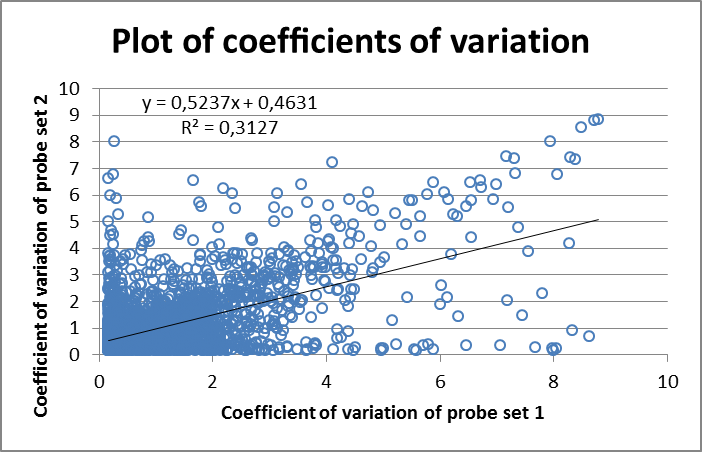 |
